# Acetyl-L-Carnitine Improves Behavior and Dendritic Morphology in a Mouse Model of Rett Syndrome

**DOI:** 10.1371/journal.pone.0051586

**Published:** 2012-12-05

**Authors:** Laura R. Schaevitz, Raffaella Nicolai, Carla M. Lopez, Stefania D'Iddio, Emerenziana Iannoni, Joanne E. Berger-Sweeney

**Affiliations:** 1 Department of Biology, Wellesley College, Wellesley, Massachusetts, United States of America; 2 Sigma Tau SpA, Pomezia, Italy; National Institute of Health, United States of America

## Abstract

Rett syndrome (RTT) is a devastating neurodevelopmental disorder affecting 1 in 10,000 girls. Approximately 90% of cases are caused by spontaneous mutations in the X-linked gene encoding methyl-CpG-binding protein 2 (MeCP2). Girls with RTT suffer from severe motor, respiratory, cognitive and social abnomalities attributed to early deficits in synaptic connectivity which manifest in the adult as a myriad of physiological and anatomical abnormalities including, but not limited to, dimished dendritic complexity. Supplementation with acetyl-L-carnitine (ALC), an acetyl group donor, ameliorates motor and cognitive deficits in other disease models through a variety of mechanisms including altering patterns of histone acetylation resulting in changes in gene expression, and stimulating biosynthetic pathways such as acetylcholine. We hypothesized ALC treatment during critical periods in cortical development would promote normal synaptic maturation, and continuing treatment would improve behavioral deficits in the *Mecp2^1lox^* mouse model of RTT. In this study, wildtype and *Mecp2^1lox^* mutant mice received daily injections of ALC from birth until death (postnatal day 47). General health, motor, respiratory, and cognitive functions were assessed at several time points during symptom progression. ALC improved weight gain, grip strength, activity levels, prevented metabolic abnormalities and modestly improved cognitive function in *Mecp2* null mice early in the course of treatment, but did not significantly improve motor or cognitive functions assessed later in life. ALC treatment from birth was associated with an almost complete rescue of hippocampal dendritic morphology abnormalities with no discernable side effects in the mutant mice. Therefore, ALC appears to be a promising therapeutic approach to treating early RTT symptoms and may be useful in combination with other therapies.

## Introduction

Rett syndrome (RTT) is a devastating neurodevelopmental disorder characterized by a period of apparently normal development lasting 6 to 18 months, followed by a rapid loss of motor and verbal ability, mental retardation and respiratory abnormalities [1], [2]. RTT is frequently caused by mutations in the X-linked gene encoding methyl-CpG-binding protein 2 (MeCP2). MeCP2 is found in the nucleus where it regulates transcription of potentially thousands of genes [3], as well as performs a variety of other functions that are not fully understood [4]. How altered expression of MeCP2 is translated into the myriad of neurochemical and neuroanatomical abnormalities that are reported in RTT remains unclear. Neurochemical abnormalities include altered concentrations of glutamate/glutamine [5], acetylcholine [6], GABA [7], and other monoamine neurotransmitters [8]; while neuroanatomical abnormalities include reduced dendritic arborization [9] and decreased spine density in cortex and hippocampus [10]. Several mouse models of RTT have been created to facilitate an understanding of underlying molecular mechanisms, and provide an avenue to test preclinical therapies (reviewed in [11]). Previously, we have shown that *Mecp2^1lox^* mutant mice, containing a deletion of exon 3 [12], exhibit many key behavioral deficits, as well as neuropathological and chemical changes observed in human cases of RTT, and provide an excellent model to test potential therapies [13], [14], [15].

Numerous therapeutic strategies have been explored in mouse models of RTT with varying degrees of success. Re-activation of *Mecp2* in null mice prevents or delays onset of symptoms depending on the timing of activation [16], [17]. Gene therapy, however, would be technically difficult in humans given that Mecp2 over-expression is as detrimental as loss-of-expression [18] and reactivation can be fatal [17]. Pharmacological treatments, including despiramine, ampakines, insulin-like growth factor-1 (IGF-1), and a partial agonist of TrkB receptors have been used to rescue respiratory abnormalities and extend lifespan following onset of symptoms in *Mecp2* mutants [19], [20], [21], [22]. Additionally, we have shown that perinatal choline supplementation improves some motor functions, but cannot restore cognitive function [23]. A survey of the literature suggests that these various pharmacological strategies improve only a subset of behavioral deficits with improvements in motor and respiratory abnormalities showing the most consistent rescue. These pharmacological interventions may fail to improve all RTT symptoms because 1) they are too selective and neglect the multitude of neurochemical imbalances, or 2) the timing of intervention is too late. Few studies assess cognitive tasks and hence virtually no studies report improved cognitive functions.

In this study, we test the efficacy of acetyl-L-carnitine (ALC), which has a broad spectrum of pharmacological effects, to ameliorate abnormalities in *Mecp2^1lox^* mutant mice. ALC improves motor and cognitive deficits in several disease models [24], [25] through a variety of mechanisms including increased acetylcholine synthesis and neurotrophin expression, as well as improved mitochondrial function [26]. Clinical trials of ALC and L-carnitine, another carnitine derivative, in older RTT girls (median ages between 6 and 10 years old) report modest improvements in sleep, energy level, communication, and cardiac function [27], [28], [29]. The modest improvements noted in these clinical trials suggest some clinical efficacy; however, the timing of the treatment may be unable to reverse the myriad of neurochemical and structural abnormalities in an already symptomatic child. We hypothesized that early treatment (during critical periods of cortical development) with ALC would be more effective at preventing the development of RTT pathology and particularly cognitive deficits; therefore, we began administering ALC at birth. Here, we report that daily injections of ALC from birth until death (postnatal day 47) improves general health, metabolic, motor, and modestly improves cognitive function in *Mecp2* mutant mice early in the course of the disease, but becomes less effective as symptoms progress. Improvements in behavior are associated with improved neuronal morphology in the hippocampus, but not with increased neurotrophin levels late in life. Importantly, we saw no adverse side effects of ALC treatment in any group.

## Materials and Methods

### Ethics Statement

Procedures were approved by the Wellesley College Institutional Animal Care and Use Committee and conformed to standards set forth in the National Institutes of Health Guide for the Care and Use of Laboratory Animals.

### Rett Mouse Model

All experiments were conducted on *Mecp2^1lox^* mice [12]. *Mecp2* ^+/−^ females, backcrossed more than 14 generations to C57BL/6J males, were bred and male and female pups were assigned to one of the following groups for experiments: male and female wildtype saline-treated (WTS and FWTS, respectively), male and female wildtype ALC-treated (WTA and FWTA, respectively), male null saline-treated (NS), male null ALC-treated (NA), female heterozygous saline-treated (FHETS) and female heterozygous ALC-treated (FHETA). After weaning, pups were housed in cages with up to 5 same sex littermates. Mice were maintained on a 12 h light/dark cycle with lights on at 7∶00 am and food and water provided *ad libitum*.

### Drug Administration

Acetyl-L-carnitine (ALC; Sigma-Tau S.p.A.; Pomezia, Italy) was administered via subcutaneous injection at a dose of 100 mg/kg of body weight. ALC was dissolved in sterile saline such that 10 μl/g body weight was given to each mouse. Control animals received an equal amount of saline. Mice were treated with ALC or saline on postnatal days (PNs) 1–47 and tested on a variety of behavioral tasks throughout the course of treatment.

### Behavioral Testing

An observer blind to the mouse's genotype performed and analyzed all behavioral tests. All mice were assessed for performance on a subset of the following behavioral tasks.

#### Neurological battery on PN 22, 30, 43

Mice were weighed daily (Male: WTS n = 6, WTA n = 6, NS n = 7, NA n = 9; Female: FWTS n = 8, FWTA n = 7, FHETS n = 7, FHETA n = 7) and a neurological battery was performed on PN 22, 30, and 43 to evaluate the general health and basic motor skills of the mice. Reaching and righting reflexes, and grip strength were measured as described previously [13]. (Male: WTS n = 6–9, WTA n = 6, NS n = 4–7, NA n = 9; Female: FWTS n = 7–8, FWTA n = 7, FHETS n = 6–7, FHETA n = 7).

#### O2 consumption/CO2 expiration on PN 22, 30, 43

An indirect calorimetry system (Qubit Systems Inc.; Ontario, Canada) was used to measure gas exchange [30]. Mice were placed in a cylindrical chamber with a fan to control chamber temperature. The chamber was connected on one side to an air pump and flow meter and on the other side to CO_2_ and O_2_ analyzers. Room air was supplied at 200 mL/min to the chamber by the pump and gas concentrations of CO_2_ and O_2_ were measured. The experimental set-up was run with no mouse in the chamber to establish concentrations of CO_2_ and O_2_ in room air. The mouse was then placed in the chamber and gas concentrations were measured over a period of 20 min (10 min for habituation and 10 minutes to determine CO_2_ and O_2_ concentrations). After 20 min the mouse was returned to its home cage. Rates of CO_2_ expiration and O_2_ consumption were calculated by finding the difference between baseline gas concentration and concentrations while the mouse was in the chamber (Male: WTS n = 4–7, WTA n = 6, NS n = 7–11, NA n = 9).

#### Dark-cycle locomotor activity on PN 21, 29, and 42

Baseline locomotion of the mouse was measured over a 12 hr dark-cycle as described previously [13]. The average number of beam breaks over the 12 hours was compared among experimental groups (Male: WTS n = 6–9, WTA n = 6, NS n = 4–7, NA n = 9; Female: FWTS n = 6–7, FWTA n = 7, FHETS n = 6–7, FHETA n = 7).

#### Accelerating rotor-rod on PN 44

Balance and motor coordination were measured on an accelerating rotor-rod (San Diego Instruments, San Diego, CA) as described previously [23]. The average of three successive trials were used for statistical analysis (Male: WTS n = 9, WTA n = 6, NS n = 7, NA n = 9; Female: FWTS n = 8, FWTA n = 7, FHETS n = 7, FHETA n = 7).

#### Object Recognition (PN28 – PN29)

Novel object memory was assessed during three sessions. This task relies on the innate tendency of a mouse to explore unfamiliar objects versus familiar objects. Testing was performed in an open-field arena as described previously [31] with the following modification. Mice were tested for short-term memory 60 min after the completion of the training session, and long-term object memory was assessed 24 hr after training. The duration of exploration (defined as the mouse's snout or forelimbs physically touching or approaching within 1 cm of an object) of familiar and novel objects was measured. A novel object discrimination index (ODI), the amount of time spent exploring the novel object over the total time exploring both novel and familiar objects, was used to measure object memory (Male: WTS n = 6, WTA n = 8, NS n = 5, NA n = 6).

#### Contextual and cued fear conditioning on PN 45

Associative learning was assessed using an automated fear conditioning system for mice (Coulbourn Instruments, Allentown, PA) with the following parameters: *Day 1, acquisition:* three min habituation to context A, followed by two tone (80 dB) and 0.5 mA footshock pairings. *Day 2, contextual retention*: five min in context A with no tone or shock. *Cued retention:* performed at least 1 hr after contextual retention consisting of three min in context B, followed by 3 min tone (80 dB). Freezing during contextual and cued retention was recorded by an automated system (Graphic State 3.0 software; Coulbourn Instruments, Allentown, PA). Prior to statistical analysis, the number of freezing intervals during acquisition, contextual and cued retention, were converted to a percentage freezing value, and baseline freezing from the acquisition phase was subtracted from freezing in the contextual phase (Male: WTS n = 6, WTA n = 6, NS n = 6, NA n = 7; Female: FWTS n = 8, FWTA n = 7, FHETS n = 7, FHETA n = 7).

### Tissue collection

Following the completion of behavioral testing on PN 47, mice were overdosed with CO_2_ and trunk blood was collected for carnitine measurements. The brains were rapidly dissected and the right hemisphere of each brain was collected for golgi analysis and the left hemisphere was dissected regionally into cerebellum, cortex, hippocampus and striatum. Brain regions were snap frozen on dry ice, and stored at −80° C for BDNF and NGF analysis.

### BNDF/NGF ELISAs

BDNF and NGF levels were measured using BDNF and NGF E_max_ immunoassay kits from Promega (Madison, WI) as described previously [15]. (Male: WTS n = 6, WTA n = 6, NS n = 6, NA n = 9).

### Golgi-Cox immunocytochemistry

Brain hemispheres collected for Golgi analysis were rinsed in physiological saline and stained using a FD Rapid GolgiStain Kit (FD Neurotech; Ellicot City, MD) according to manufacture's instructions. Brains were cut into 125 µm thick sections on a vibratome, mounted on slides, and developed. Neurons, in which the whole dendritic tree was easily distinguished from surrounding neurons, were selected at random from the suprapyramidal blade of the dentate gyrus for analysis. A stack of images every 15 µm through the section were acquired at 20X on a Nikon 80i microscope. The cell body and dendritic arbors were traced using Photoshop (Adobe; San Jose, CA) and imported into Image J (Maryland, USA) to calculate cell body size and dendritic length. For Sholl analysis, an image of concentric circles at 15 µm intervals was created and superimposed over the dendritic arbor to determine the number of crossings every 15 µm from the cell body. (Male: WTS, WTA, and NS, n = 37–38 neurons from 6 mice; NA, n = 54 neurons from 9 mice).

### Carnitine Measurements

The serum samples were shipped to Sigma-Tau on dry-ice and were stored there at −80°C until analysis (Male: WTS n = 6, WTA n = 6, NS n = 7, NA: n = 9). Twenty microliters of serum sample were deproteinized with 80 microliters of methanol containing internal standard. Then the mixture was vortexed and centrifuged at 10000 RPM for 10 min. The clear supernatant was injected onto the HPLC system.

The samples were quantified using a reference standard curve, to determine L-carnitine (LC), acetyl-L-carnitine (ALC) and propionyl-L-carnitine (PLC) levels, respectively. Acetyl-d3-L-carnitine was used as internal standard. The analysis of LC, ALC and PLC were performed by HPLC (Waters Alliance 2695) equipped by a column Hypersil APS2 250×4.6 mm 5 µm Thermo (CPS) and detection by Mass Spectrometry (Waters Micromass ZQ 2000) with masses set at 162.06, 204.11, 218.16 and 207,22 Da., with ion-source operating in positive ion-mode.

### Statistical Analysis

Data were analyzed using two-way analysis of variance (ANOVAs) using genotype and treatment as the between group factors. Data collected over multiple days were analyzed using two-way repeated-measure ANOVAs with testing day as the repeated measures. Post hoc bonferroni analyses (minimizes error due to multiple comparisons) were used to examine differences between groups following ANOVAs. One sample t-tests were used to determine whether the object discrimination index calculated in the object recognition task was statistically different from 0.5, which would denote chance or no preference. All analysis were performed using SPSS software (SPSS Inc. Chicago, IL, USA) with p<0.05 considered significant.

## Results

### Levels of carnitines are significantly elevated in wildtype and null ALC-treated mice

To ensure that daily injections of acetyl-L-carntine (ALC) effectively increased levels of carnitines in the mice, levels of L-carnitine (LC), ALC, and propionyl carnitine (PLC) were measured in blood plasma collected at death. Both wildtype and null groups treated with ALC had significantly higher levels of LC, ALC, and PLC than saline-treated groups [ANOVA effect of treatment: LC [µM]: [*F*_(1,27)_ = 109.23, *p*<0.001]; ALC [µM]: [*F*_(1,27)_ = 216.38, *p*<0.001]; PLC [µM]: [*F*_(1,27)_ = 227.75, *p*<0.001]; see Table 1]. Previously, carnitine deficiency has been implicated in a subset of RTT cases and symptomology [28]. We found no significant differences between saline-treated WT and saline-treated null mice on any carnitine measurements suggesting that carnitine deficiency in adulthood is unlikely to contribute directly to symptomology in this RTT mouse model. Nevertheless, it is clear that the daily subcutaneous ALC injections were effective in increasing blood carnitines in the mice and it is likely that this effect was apparent from PN 1 when injections began.

**Table 1:** Carnitine concentrations in blood plasma collected at PN 47.

	Free Carnitine [µM]	Acetyl Carnitine [µM]	Propionyl Carnitine [µM]
**WTS**	31.6±3.3	25.3±2.0	0.91±0.04
**WTA**	100.1±7.3*	308.4±7.9*	3.38±0.15*
**NS**	23.6±3.9	36.1±7.8	0.51±0.07
**NA**	115.4±9.7*	327.4±27.9*	2.32±0.18*

All values represent mean ± SEM. The * indicates values significantly different from WTS values. WTS: wild type saline-treated; WTA: wild type ALC-treated; NS: null saline-treated; NA: null ALC-treated.

### ALC-treatment improves general health in Mecp2-mutant mice

**Figure 1:**
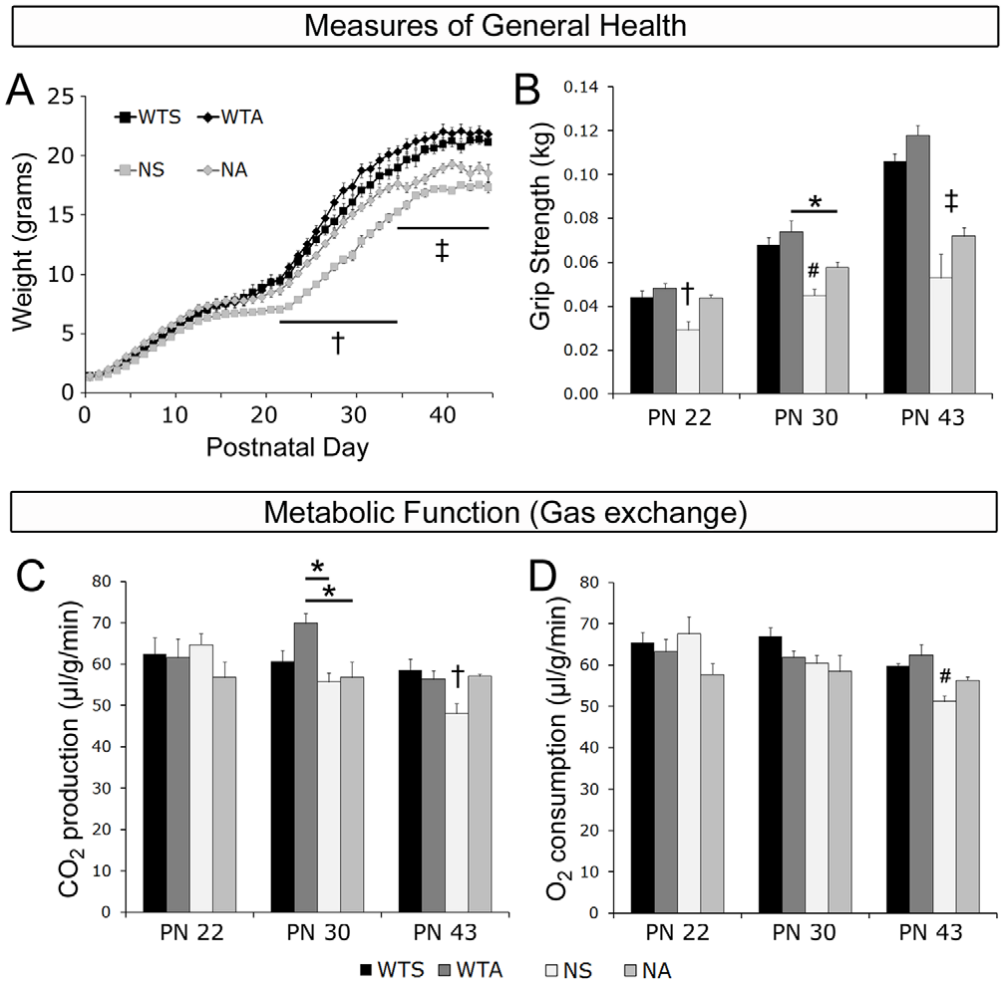
General health and metabolic function are improved in ALC-treated null mice compared to saline-treated nulls. (**A**) Weight (g) was measured daily between postnatal day (PN) 1 and PN 45 in all mice. Saline-treated nulls weighed significantly less than all other groups between PN 22 and 35 (*p*'s<0.05). Between PN 36 and 45, both saline-treated and ALC-treated null mice weighed significantly less than saline-treated and ALC-treated WT mice (*p*'s<0.05). (**B**) Forepaw grip strength (kg) was assessed on PN 22, PN 30, and PN 43. Grip strength was lower in saline-treated nulls compared to saline-treated WTs on all PN's examined (*p*'s<0.003). In ALC-treated nulls, grip strength was improved to saline-treated WT levels on PN 22, intermediate between saline-treated null and saline-treated WT levels on PN 30, and significantly impaired compared to saline-treated WT mice on PN 43. Metabolic function was assessed by measuring carbon dioxide (CO_2_) production (**C**) and oxygen (O_2_) consumption (**D**) on PN 22, PN 30, and PN 43. CO_2_ and O_2_ levels were significantly decreased in saline-treated null mice compared to both WT groups (saline-treated and ALC-treated) on PN 43 (*p*'s<0.03). CO_2_ levels were significantly improved in ALC-treated nulls compared to saline-treated null mice (*p*<0.005) and O_2_ levels were intermediate between saline-treated null and saline-treated WT levels on PN 43. All values represent the mean ± SEM. WTS: wild type saline-treated; WTA: wild type ALC-treated; NS: null saline-treated; NA: null ALC-treated. † NS vs. WTS, WTA, NA (*p*<0.05) # NS vs. WTS, WTA (*p*<0.05) ‡ NS and NA vs. WTS and WTA (*p*'s<0.05) * *p*<0.05 between indicated groups.

Previously, we have shown that general health is impaired in *Mecp2* null mice. The mutant mice have reduced body size and weak forepaw grip strength [13]. In the current study, mice were weighed daily from PN 1 until PN 47 (Figure 1A). The weights of saline-treated WT mice increased steadily across the 47 days measured; there were two periods of relatively slow weight gain between PN 15 and PN 25 and between PN 35 and PN 47. A two-way repeated-measures ANOVA revealed a significant genotype by treatment interaction for weight across days [*F*_(9,15)_ = 3.537, *p* = 0.015]. ALC treatment did not affect weight in wild type mice on any postnatal day [saline-treated vs. ALC-treated WTs, all *p*'s>0.05]. Null saline-treated mice, however, gained weight more slowly starting just after weaning (PN 22 until death) relative to both saline-treated and ALC-treated WT mice [all *p*'s<0.02]. Similar to wild types, weight gain in saline-treated null mice leveled off albeit at a lower overall level than controls around PN 36. ALC treatment significantly improved weight gain in null mice between PN 22 and 35 [saline-treated vs. ALC-treated nulls; all *p*'s<0.02]. While ALC-treated null mice were always numerically heavier than saline-treated null mice, there was no significant difference in weight between null groups after PN 36 and until death [all *p*'s>0.20] with both groups of null mice weighing significantly less than both groups of WT mice [all *p*'s<0.001].

Reduced forepaw grip strength is another consistent phenotype of *Mecp2* null males [13], [23]. We assessed grip strength longitudinally at three points corresponding to early (PN 22), middle (PN 30), and late (PN 43) times during the progression of symptoms. On all three postnatal days examined, grip strength differed both by treatment and by genotype using two-way ANOVAs [*F*'s_(1,24)_>7.7, *p*'s<0.01] (Figure 1B). Posthoc analysis revealed that, on all three days, grip strength in saline-treated WT males was significantly higher than in saline-treated null males [*p*'s<0.003]. While ALC-treatment did not affect grip strength in WT males on any day, ALC treatment did significantly improve grip strength in ALC-treated nulls as compared to saline-treated nulls on PN 22 [*p* = 0.004]; there was a trend toward improvement on PN 30 [*p* = 0.067], however, grip strength was not improved on PN 43.

As a measure of metabolic function, we assessed gas exchange by measuring carbon dioxide (CO_2_) production and oxygen (O_2_) consumption again at three points across the progression of symptoms. In males, there was a significant interaction of genotype and treatment for CO_2_ expired across the days of testing [repeated-measures ANOVA: *F*_(2,20)_ = 4.07, *p* = 0.03] (Figure 1C). There were no significant differences among the four groups on PN 22 and PN 30. However, on PN 43, saline-treated WT males expired significantly more CO_2_ than saline-treated null mice [*p* = 0.012]. ALC treatment improved CO_2_ expiration; ALC-treated nulls expired significantly more CO_2_ than saline-treated nulls [*p* = 0.005]. In fact, ALC-treated null mice were virtually indistinguishable from saline-treated WTs across the days of testing. O_2_ consumption varied by treatment across days of testing [repeated-measures ANOVA: *F*_(2,20)_ = 4.8, *p* = 0.02], and on PN 43 varied by genotype [ANOVA: *F*_(1,24)_ = 15.73, *p* = 0.001]. O_2_ consumption was similar across the three days measured in both groups of WT mice. For saline-treated null mice, however, O_2_ consumption was significantly lower on PN 43, signaling an onset of metabolic dysfunction [saline-treated WTs vs. saline-treated nulls; *p* = 0.03] (Figure 1D). This dysfunction was not evident on PN 43 in the ALC-treated null mice [saline-treated WTs vs. ALC-treated nulls; *p* = 0.177], suggesting that ALC treatment forestalled some of the metabolic abnormalities in the null mice. Based on our visual observations along with weight, grip strength, and gas exchange data, null saline-treated mice begin to show physical abnormalities around weaning (PN 21). The ALC-treated null mice were healthier with better maintenance of coat fur and metabolic function than were saline-treated null mice until later (around PN 35).

### Motor function is improved in Mecp2 null mice during early treatment with ALC

**Figure 2:**
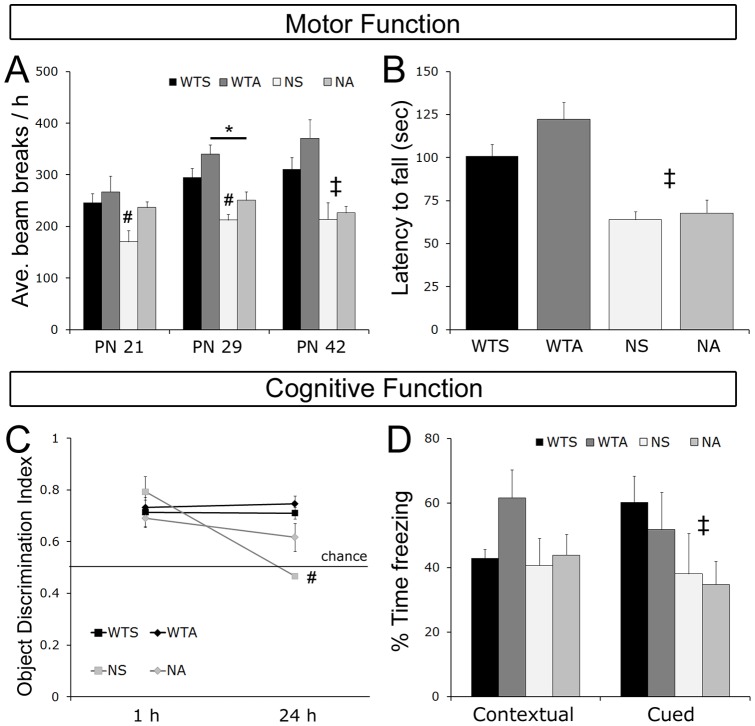
Motor and cognitive functions are modestly improved in null ALC-treated mice compared to saline-treated null mice during early treatment. General activity levels (**A**) were assessed at PN 21, PN 29, and PN 42. Saline-treated null mice were hypoactive compared to saline-treated wild type mice at all ages (*p*'s<0.05). Activity in ALC-treated null mice increased to a level intermediate between saline-treated WT and null mice on PN 21 and 29 (*p*'s>0.05), but was significantly impaired on PN 43. Deficits in motor coordination (**B**), assessed at PN 44, were unaffected by ALC treatment in null mice. Cognitive function was measured at PN 28 using an object recognition task (**C**). Object memory was intact in all mice 1 h after object training and in both wild type groups 24 h after training. Saline-treated nulls exhibited no memory for the object 24 h after training, while object memory in ALC-treated null mice was intermediate between saline-treated null and saline-treated WT levels. Cognitive function was measured on an associative fear conditioning task at PN 46 (**D**). All groups performed the contextual fear portion of the task similarly, while both groups of null mice were impaired on the cued fear portion of the task. ALC treatment did not affect performance on either portion of the task. All values represent the mean ± SEM. ODI: object discrimination index; WTS: wild type saline-treated; WTA: wild type ALC-treated; NS: null saline-treated; NA: null ALC-treated. † NS vs. WTS, WTA, NA (*p*<0.05) # NS vs. WTS, WTA (*p*<0.05) ‡ NS and NA vs. WTS and WTA (*p*'s<0.05) * *p*<0.05 between indicated groups.

Motor function is severely impaired in *Mecp2* null mice [13]. In this study, we performed a longitudinal assessment of general motor activity levels during the dark cycle early (PN 21), midway (PN 29), and late (PN 42) in the progression of symptoms. Additionally, we evaluated motor coordination using the rotorod at PN 44 (late). There was a significant difference in baseline locomotor activity among the groups by genotype on each day tested [ANOVA's: *F*'s _(1,24)_>7.3, *p*<0.01]. In both groups of WT mice, locomotor activity numerically increased from PN 21 to PN 42 (Figure 2A). However, this pattern was not evident in either group of null males where locomotor activity levels were relatively steady across the days of testing. There was a significant effect of ALC treatment on PNs 21 and 29 [ANOVA: *F*'s_(1,27)_ >4.9, *p*<0.035]. Post hoc analysis on PN 21 and PN 29 showed that saline-treated null mice were hypoactive compared to saline-treated WTs on both days [*p*'s>0.05]. Locomotor activity levels in ALC-treated null mice were in beween saline-treated WT and saline-treated null mice, two groups that were significantly different from each other. The ALC-treated nulls exhibited activity levels that never were significantly lower than saline-treated WTs [*p*'s>0.28]; the ALC-treated nulls also never had significantly higher levels of locomotor activity than saline-treated nulls [*p*'s>0.13]. These data suggest that ALC led to an improvement in locomotor activity on PN 21 and 29 in the null mice, but did not increase activity fully to WT levels.

Motor coordination (as measured by rotorod performance) was significantly impaired in *Mecp2* null mice at PN 44, similar to previous reports [23]. Both groups of wildtype mice performed better than either group of null mice [ANOVA: *F*_(1,27)_ = 26.768, *p*<0.001, Figure 2B] and there was no significant effect of ALC treatment [ANOVA: *F*_(1,27)_ = 1.177, *p* = 0.29]. Post-hoc comparisons revealed that ALC did not improve motor coordination in either the WT or the null males on PN 44 [*p*'s>0.23]. Altogether, this longitudinal assessment of motoric ability suggests that, during early treatment with ALC, motor function is improved in null mice, but by PN 42, ALC has no effect on motor deficits in null males.

### Cognitive function is partially improved in Mecp2 null mice early, but not late in life with ALC treatment

Ideally, a therapy would ameliorate the severe cognitive deficits associated with RTT in addition to improving general health, metabolic, and basic motor function. Given that some of the networks that support cognition are formed during critical periods in cortical development around the time of birth [32], we hypothesized that starting ALC treatment at birth would have the best chance of rescuing cognitive deficits that manifest later in life. Therefore, we assessed the ability of ALC to reverse cognitive deficits early (using novel object recognition at PN 28) and late (using associative fear conditioning at PN 45) in the progression of symptoms. On the object recognition task, short-term memory (1 h after object training) was intact in all the mice, including saline-treated null mice. At 1 h after object training, all four groups spent significantly more time with Object 2 (the novel object) than with Object 1 (object presented during object training). The object descrimination index (ODI: see materials and methods for calculation) for all groups was significantly greater than chance –50% levels [One-sample t-test: all *p*'s<0.016; Figure 2C], indicating that all the mice were able to acquire and maintain a memory of an object for an hour. At 24 h, there was a significant effect of genotype [ANOVA: *F*_(1,23)_ = 23.798, *p*<0.001] and a significant effect of treatment [ANOVA: *F*_(1,23)_ = 5.927, *p* = 0.02] on long-term object recognition memory. WT saline-treated and WT ALC-treated mice spent significantly more time with Object 3 (novel object), indicating long-term memory for Object 1 [ODI's significantly higher than chance *p*'s<0.001]. Null saline-treated mice exhibited no memory for Object 1 [ODI not significantly different than chance p = 0.597]. ALC-treated nulls showed a trend toward a preference for the novel object [*p* = 0.08]. Post-hoc analysis indicated that WT saline-treated and WT ALC-treated mice performed significantly better than saline-treated nulls at 24 h [both *p*'s<0.05]. The performance of the ALC-treated null group was intermediate between the WT and saline-treated null groups, which were significantly different from each other [p<0.001]. These data suggest that ALC led to a modest improvement in the nulls performance on this cognitive task on PN 29, though their performance was not improved to WT levels. Differences in performance on the object recognition task were not due to a difference in the amount of time spent with the objects during training or choice sessions. Two-way ANOVAs revealed no significant effects of genotype, treatment, and no genotype-treatment interactions for amount of time spent with objects during any of the sessions (data not shown).

An improvement in cognitive function was not evident after ALC treatment on the fear conditioning task at PN 45. Similar to our previous studies [13], *Mecp2* null mice displayed deficits on the cued (significant effect of genotype [ANOVA: *F*_(1,22)_ = 4.51, *p*<0.001], Figure 2D), but not contextual fear conditioning portion of the associative fear conditioning task [effect of genotype ANOVA: *F*_(1,22)_ = 2.813, *p* = 0.11]. We found that performance varied considerably within groups, which made clear differences among the groups difficult to tease out. Nevertheless, posthoc analysis indicate that both WT saline-treated and ALC-treated mice performed the cued task better than null saline-treated or null ALC-treated males. There were no significant effects of treatment nor any significant genotype x treatment effects on either the cued [ANOVA: *F's*_(1,22)_>0.484, *p*>0.494] or contextual [ANOVA: *F's*_(1,22)_>0.913, *p*>0.35] portion of this cognitive task indicating that ALC did not improve associative memory formation on PN 45.

### General health, motor and cognitive functions are mostly normal in Mecp2 heterozygous females

**Figure 3:**
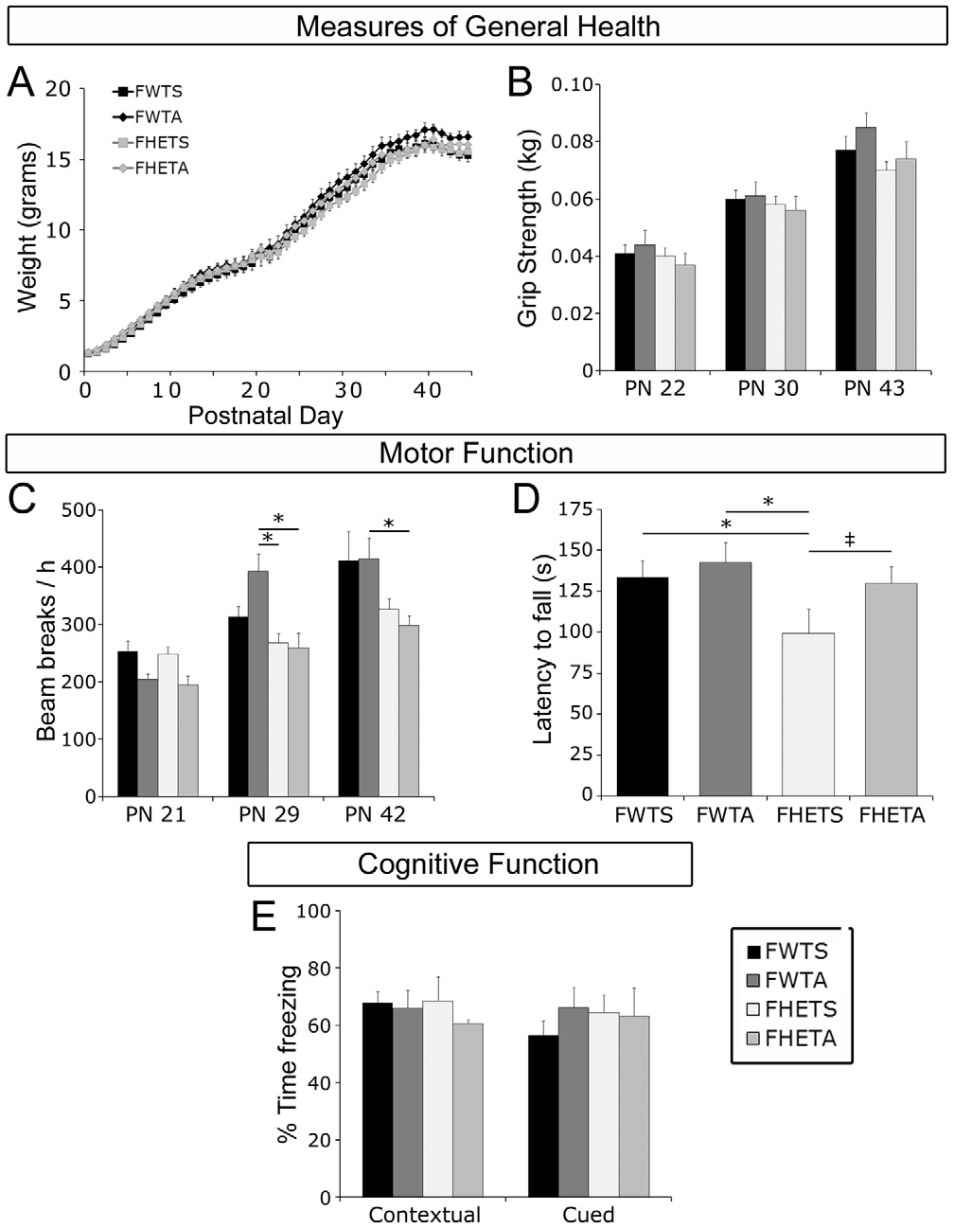
General health, motor and cognitive functions are predominately normal in *Mecp2* heterozygous females at the ages examined. Weight (**A**) and grip strength (**B**) were not statistically different between the four groups of female mice. Locomotor activity (**C**) measured over the 12 hour dark cycle was similar between saline-treated WT females and both groups of heterozygous females (saline and ALC-treated). ALC-treated WT females were significantly more active than heterozygous females (saline and ALC-treated), but not saline-treated WT females on PN 29 and PN 42. Motor coordination measured on the rotorod at PN 43 (**D**) was significantly impaired in saline-treated heterozygous females compared to WT females (saline-treated and ALC-treated). ALC treatment improved performance of heterozygous females to a level intermediate between saline-treated heterozygous and WT females. Performance on both contextual and cued fear conditioning (**E**) was similar between all four groups of female mice at PN 46.

In contrast to *Mecp2* null male mice, *Mecp2* heterozygous females do not exhibit poor health (weight or grip strength) relative to control females between birth and PN 47, consistent with an onset of most symptoms after 6 months of age [13], [23]. Two-way repeated measures ANOVAs revealed no significant effects of genotype, treatment, or genotype x treatment interactions on weight gain (between PN 1 and PN 47; Figure 3A) or forepaw grip strength (measured on PN 22, PN 30, and PN 43; Figure 3B). All four groups of female mice were statistically indistinguishable.

Deficits in motor function, including locomotor activity and motor coordination, can appear after PN 35 in *Mecp2* heterozygous females [13], [23]. Baseline locomotor activity varied significantly by genotype and by treatment across the days of testing [ANOVA's: *F*'s _(2,21)_>3.5, *p*'s*<*0.05; Figure 3C]. Posthoc analysis revealed that these effects were primarily because ALC-treated WT females were hyperactive relative to ALC-treated and saline-treated heterozygous females on PN's 29 and 42 [*p*'s<0.02]. Locomotor activity levels were not significantly different between WT saline-treated and either group of heterozygous female mice [saline or ALC-treated; *p*'s>0.05] on any day of testing. ALC treatment did not affect performance of the heterozygous females. For motor coordination, assessed at PN 44 on the rotorod, a one-way ANOVA revealed a significant effect of genotype [ANOVA: *F*_(1,24) = _8.1, *p = *0.009] and treatment [ANOVA: *F*_(1,24) = _6.3, *p = *0.02] on latency to fall (Figure 3D). Latency to fall on the rotorod was significantly shorter, indicating impaired motor coordination, in saline-treated heterozygous females as compared to both saline-treated and ALC-treated WT females [*p*'s<0.025]. In contrast, performance of ALC-treated heterozygous females was indistiguishable from both WT groups (saline and ALC-treated) and there was a trend toward an improvement in motor coordination as compared to saline-treated heterozygous females [*p* = 0.055] suggesting that ALC-treatment improves motor coordination very modestly in *Mecp2* heterozygous females.

Performance on an associative fear conditioning tasks at PN 45 was similar amongst all groups (Figure 3E). There were no significant effects of genotype or treatement on either the contextual or cued portion of this cognitive task suggesting that heterozygous females are not impaired cognitively at this age. Due to paucity of behavioral impairments noted in heterozygous females and the need for their use for breeding, analysis of ALC's effects on brain neurochemistry and neuroanatomy were only carried out in the males.

### ALC treatment does not alter BDNF or NGF expression in males

**Figure 4:**
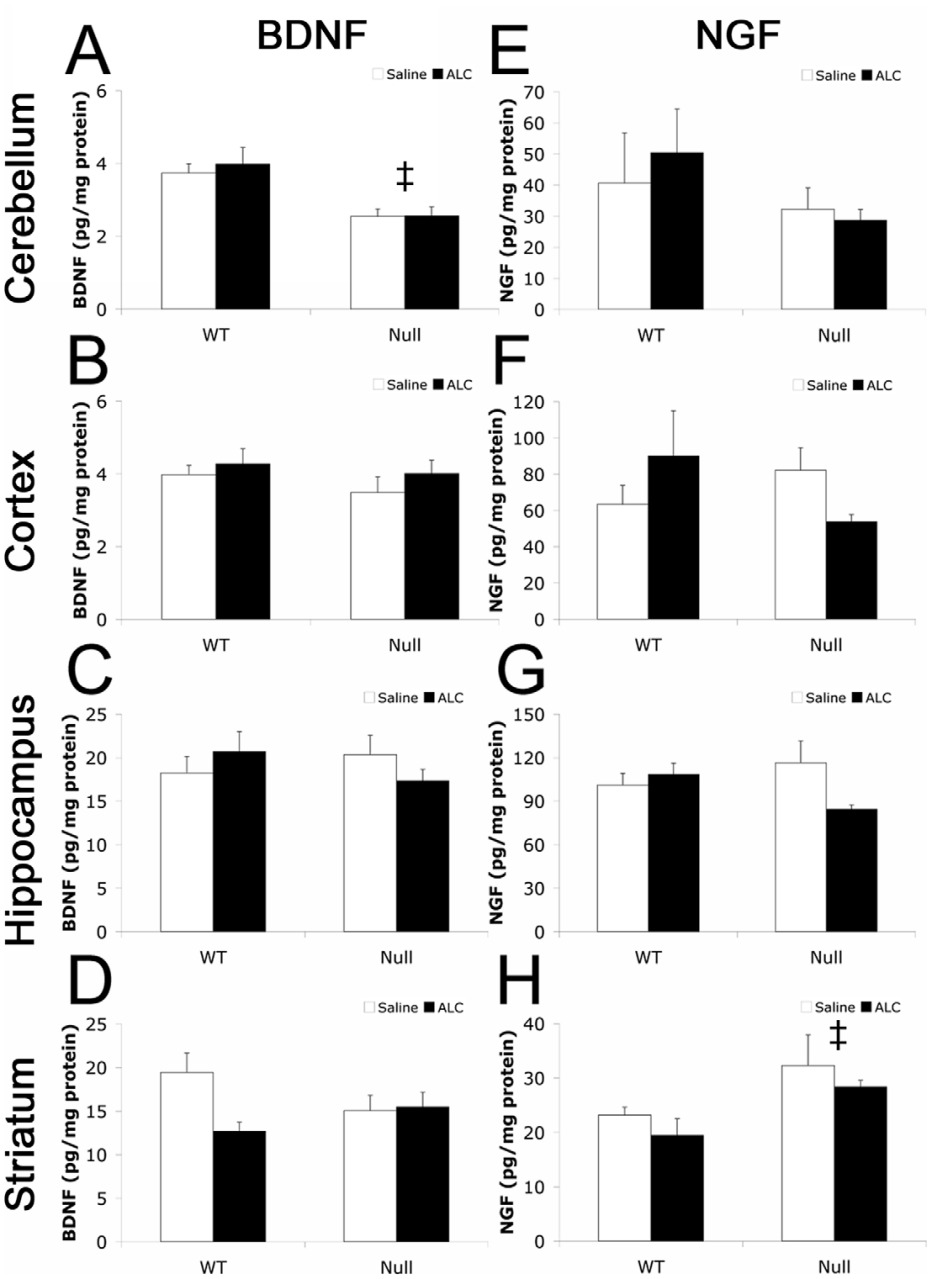
ALC treatment does not affect BDNF or NGF levels at PN 47. Levels of BDNF (**A**–**D**) and NGF (**E**–**H**) were measured by ELISA in four brain regions: cerebellum (**A,E**), cortex (**B,F**), hippocampus (**C,G**), and striatum (**D,H**). BDNF levels were significantly reduced in null compared to WT mice in the cerebellum (**A**) [*F*_(1,26)_ = 11.147, *p*<0.001] and NGF levels were significantly elevated in the striatum in null groups compared to WT mice (**H**) [*F*_(1,22)_ = 7.067, *p* = 0.016]. There were no significant effects of ALC treatment on either BDNF or NGF levels. All values represent the mean ± SEM. WTS: wild type saline-treated; WTA: wild type ALC-treated; NS: null saline-treated; NA: null ALC-treated. ‡ NS and NA vs. WTS and WTA (*p*'s<0.05).

Improvements in cognitive functioning following treatment with ALC have been linked to changes in NGF expression [33]. Because MeCP2 regulates BDNF expression *in vivo* [34] and behavioral performance is associated with neurotrophin levels in functionally relevant brain regions [35], [36], we predicted that improvements in general health and behavior in ALC-treated null mice, compared to saline-treated null mice, may be associated with altered NGF and possibly BDNF expression. We examined the levels of BDNF and NGF after behavioral testing was completed (PN 47). As we expected, there were significant effects of genotype on BDNF (Figure 4A–D) and NGF (Figure 4E–H) trophic factor levels. In the cerebellum, there was a significant reduction in the level of BDNF in null mice (both saline and ALC-treated) compared to WT mice (both saline and ALC-treated) [ANOVA: *F*_(1,26)_ = 11.147, *p*<0.001]. We found no significant effect of genotype on BDNF levels in any other brain region examined. In the striatum, there was a significant increase in the level of NGF in null mice (null saline-treated and ALC-treated combined) compared to WT mice (WT saline-treated and ALC-treated combined) [ANOVA: *F*_(1,22)_ = 7.067, *p* = 0.016]. Contrary to our prediction, we found no significant effects of ALC treatment or significant interactions between genotype and treatment on either BDNF (Figure 3A–D) or NGF levels (Figure 3E–H) in different brain regions.

### ALC rescues dendritic abnormalities in the hippocampus of Mecp2 null mice

**Figure 5:**
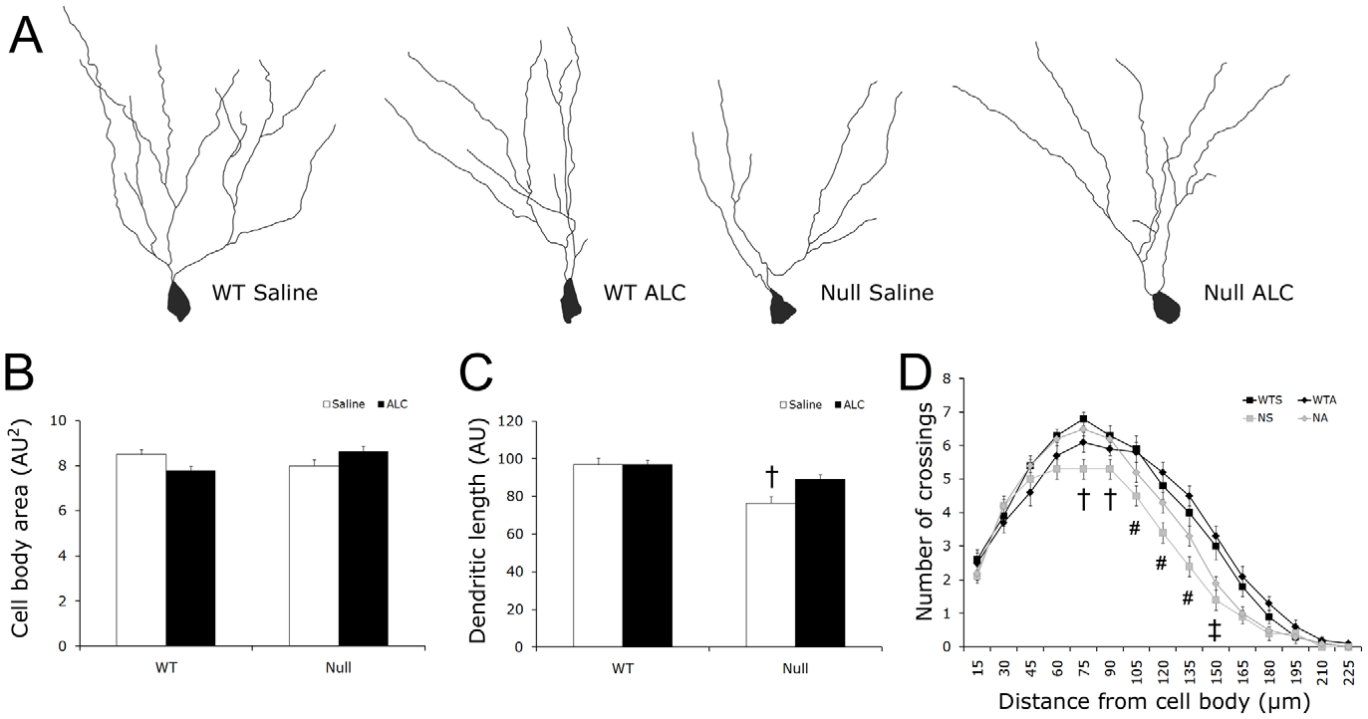
Reduced dendritic length and complexity are mostly rescued in ALC-treated null mice. Representative drawings of Golgi-Cox stained neurons analyzed in the dentate gyrus of the hippocampus in (**A**) WT saline-treated, WT ALC-treated, null saline-treated, and null ALC-treated mice. (**B**) Area of the cell body was not affected by genotype or treatment. (**C**) Total dendritic length was significantly reduced in saline-treated nulls compared to both WT groups (*p*'s<0.001) and was rescued with ALC treatment in null mice (saline-treated vs. ALC-treated nulls; *p* = 0.016). (**D**) Dendritic complexity was decreased in saline-treated null mice compared to both groups of WT mice indicated by fewer dendrite crossings between 75 and 150 µm from the cell body. ALC treatment in null mice had a significant effect on the number of dendritic crossings in close proximity to the cell body. All values represent the mean ± SEM. AU = arbitrary units. WTS: wild type saline-treated; WTA: wild type ALC-treated; NS: null saline-treated; NA: null ALC-treated. † NS vs. WTS, WTA, NA (*p*<0.05) # NS vs. WTS, WTA (*p*<0.05) ‡ NS and NA vs. WTS and WTA (*p*'s<0.05).

Reduced dendritic size and complexity as well as smaller cell body size are common in cortex and hippocampus of RTT individuals [37], [38], [39], [40], and are likely associated with impaired cognitive function [41], [42]. Alterations in dendritic arborization have been noted in *Mecp2* mutant mice in cortex and hippocampus previously [38]. We analyzed cell morphology in the dentate gyrus of the hippocampus (cell body size and dendritic length) and performed a Sholl analysis to measure dendritic complexity (Figure 5A). Although Golgi-impregnation, used in these analyses, does not label every cell, cursory analysis indicated that similar numbers and types of neurons were labeled in all groups of mice. For cell body size, there was a significant genotype by treatment interaction [ANOVA: *F*_(1,160)_ = 7.75, *p* = 0.006], but no significant genotype or treatment effect alone (Figure 5B). This interaction was due to the fact that, in ALC-treated WT brains, cell body size was slightly smaller than in saline-treated WT, however, in null ALC-treated brain, cell body size was slightly larger than in null saline-treated mice. However, there were no significant within-genotype differences. Dendritic length varied by genotype [ANOVA: *F*_(1,159)_ = 20.45, *p*<0.001] and by treatment [ANOVA: *F*_(1,159)_ = 4.13, *p* = 0.044] and there was a significant interaction between genotype and treatment [ANOVA: *F*_(1,159)_ = 4.19, *p* = 0.042, Figure 5C]. Dendritic length was longer in dentate granual neurons of saline-treated WTs as compared to saline-treated nulls [*p*<0.001]. ALC treatment was associated with significantly increased dendritic length in ALC-treated nulls as compared to saline-treated nulls [*p* = 0.016]. In addition, a two-way repeated measures ANOVA showed a significant effect of genotype [ANOVA: *F*_(14,150)_ = 3.17, *p*<0.001], (Figure 5D) on dendritic complexity. Posthoc analysis indicated that saline-treated WTs had significantly more dendritic crossings between 75 µm and 150 µm from the cell body than saline-treated null mice [*p*'s<0.05]. ALC-treated null mice exhibited a more complex dendritic structure similar to both WT groups, which was especially evident proximal to the cell body (75 µm and 90 µm) where ALC-treated null mice had significantly more dendritic crossings that saline-treated nulls [*p*'s<0.05].

## Discussion

### Rett-like behavioral deficits are improved early but not late by ALC treatment

ALC treatment, which significantly elevates carnitine levels in the blood, delays the onset of Rett-like symptoms in male *Mecp2^1lox^* mice. Daily treatment with ALC significantly improves forepaw grip strength on PN 22, weight gain until PN 35, and prevents metabolic abnormalities that develop by PN 43. In addition, ALC treatment in *Mecp2* null males improves locomotor activity levels on PN 21 and PN 29, and partially attenuates cognitive deficits on an object recognition task at PN 28. Though ALC may delay the onset of some behavioral deficits, it does not significantly improve performance on behavioral tasks assessed later in life, for example locomotor activity (PN 42), rotorod (PN 44) or associative fear conditioning (PN 45). In *Mecp2* heterozygous females, ALC treatment improves performance on the rotorod (PN 44); the only behavioral task in which saline-treated heterozygous females exhibited significant impairments at the age tested. Thus, ALC may be able to forestall the onset of Rett-like symptoms; nevertheless, towards the end of life, ALC is not sufficient to prevent symptoms from occurring. The reduced effectiveness of ALC treatment on performance during the later stages of the disease may be due either to ALC's inability to counteract increasingly severe neurochemical changes in RTT [43], [44], a reduction in responsivity of the brain to ALC as the animal ages [45], or possibly reduced sensitivity to the effects of ALC with time.

### Dendritic morphology and possible neurochemical correlates of behavioral improvements

In the adult, dendritic branching patterns play a critical role in neuronal function [46] where reduced dendritic complexity is often associated with disease pathology [41], [42]. In RTT, dendritic length and complexity are reduced in key functional brain regions such as cortex and hippocampus [37], [38]. Here we show that ALC has significant long-term effects on hippocampal neuronal morphology; both dendritic length and complexity are significantly improved in ALC-treated male mutant mice. Dendritic branching patterns are established through a process of dendritic extension, synapse formation, and stabilization that is dynamic early in development and has relatively little plasticity as arbors mature [47]. Therefore, improvements in dendritic structure are likely associated with ALC administration early during postnatal development. This substantial rescue of hippocampal dendritic morphology is likely responsible for improvements on the hippocampal-dependent object recognition task assessed on PN 28 [48].

Early behavioral recovery following administration of ALC, in this study, may be derived from one or more of ALC functions including increased mitochondrial energy production, increased synthesis and release of acetylcholine, glutamate, dopamine, and GABA, increased NGF levels and the number of its receptors, or modulation of histone acetylation and associated gene transcription ([26] and references therein). Improvements in behavior and neuronal morphology, which both can be modulated by activity-dependent changes in neurotrophin expression ([49], [50], reviewed in [51]), led us to examine whether ALC's effects are mediated by altering BDNF or NGF levels. Contrary to our prediction, neurotrophin levels measured at PN 47 are not significantly different between saline and ALC-treated groups. Additionally, we were surprised that, with the exception of the cerebellum, BDNF levels are not decreased in the null mice. In *Mecp2* mutants, BDNF levels are frequently reduced across a number of brain regions [15], [20]. Thus, the discrepancy between BDNF levels here and previously reported may be associated with the number of behavioral tasks these mice performed immediately prior to neurotrophin analysis (locomotor activity PN 42, metabolic activity PN 43, rotorod PN 44, and fear conditioning PN 45–46). Previous studies show that exercise and learning, both of which occurred during behavioral testing, can increase BDNF levels [52], [53].

In addition to neurotrophins, there are other mechanisms through which ALC could exert its positive effects on behavior. RTT is associated with alterations in several neurotransmitters including acetylcholine and glutamate [6], [54], [55]. Administration of ALC can stimulate the synthesis and release of acetylcholine [56], which may be associated with improvements in both motor and cognitive function in ALC-treated mutants [57], [58]. Furthermore, acetylcholine is a critical morphogenic signal in developing cortical networks that support cognitive function throughout life [59]. ALC is also incorperated into glutamate during synthesis [60]. Following long-term administration, ALC can increase glutamate levels [61] and prevent age-associated declines in the number of NMDA glutamate receptors [62], [63] that are believed to play a critical role in learning and memory [64]. Thus cognitive improvements in our ALC-treated *Mecp2* null mice may also be explained by enhanced cholinergic and glutamatergic signaling during critical periods in development.

The critical question is why ALC-related improvements in behavior are not sustained as symptoms progress. We can speculate on a number of possibilities: 1) the high affinity carnitine transporters required for movement of ALC into the brain [65] may downregulate with daily injections, 2) in the case of cognitive function, the test choosen to assess cognitive deficits early (i.e. object recognition) is more sensitive to ALC-related improvements in performance than the later task (associative fear conditioning), or 3) the neurochemical changes induced by ALC may be critical for establishing neuronal networks but not for maintaining synaptic signaling after symptoms progress. Further experiements, will help us to distinguish among these plausible alternatives and provide insights into how to sustain these improvements as symptoms progress.

### Effective therapies in treating Rett syndrome

In symptomatic RTT girls, reduced neuronal connectivity [38], [66] and deficits in both excitatory and inhibitory synaptic transmission [67], [68] likely underlie the majority of health, motor, respiratory, cognitive, and social abnormalities. Expression of Mecp2 in postmitotic neurons early during the period of synaptic refinement [69], [70], as well as later activity-dependent regulation of Mecp2 function [71], [72] places it in a position to control critical aspects of both brain maturation and adult function. In this study, treatment with ALC was initiated at birth in an attempt to rescue abnormalities in brain pathology including defects in dendritic arborization that are established during the first weeks of life [49], [73]. In support of the fact that this is a realistic possibility, we are able to rescue most abnormalities in hippocampal dendritic morphology with early ALC treatment. Improvements in morphology demonstrated here are more extensive than those in other theraputic strategies [19], [74]. Treatment of *Mecp2* mutant mice with IGF−1 significantly increases spine density (with no mention of improvements in dendritic arbor; [19]) and reintroduction of *MeCP2* into *Mecp2* null mouse cells in culture ameliorates deficits in dendritic length and complexity [74]. The current study, however, is the first to report significant improvements in dendritic arborization *in vivo*. The essential nature of the dendritic arbor as a scaffold for synaptic contacts between neurons suggests that, although continued treatment with ALC appeared unable to ameliorate synaptic signalling deficits that develop later in the course of the symptoms, the underlying structure and connectivity to support improved cognitive function are present. These studies support an essential role for Mecp2 in synaptic function in the adult. ALC-treatment in *Mecp2* mutant mice appears to lead to a more normal developmental trajectory, both behaviorally and anatomically, until around the time coincident with closure of critical periods in cortical development. The delay in motor and cognitive dysfunction in *Mecp2* nulls treated with ALC, therefore, may be more analogous to the later onset of motor and cognitive deficits displayed in *Mecp2^308^* mutant mice (containing a late truncation of *Mecp2*), in which dendritic arbors are normal but synaptic function is disrupted [75]. These findings are also consistent with experiments in which Mecp2 is inactivated in adult mice and mutants exhibit behavioral abnormalities statistically indistinguishable from those demonstrated in mice lacking Mecp2 function throughout life [76]. It may be possibile that rescuing synaptic function in the adult, despite abnormal gross neuronal connectivity, will be sufficient to ameliorate symptoms in *Mecp2* mutants. Certainly, several studies demonstrate that reactivation of Mecp2 in fully symptomatic mutants is capable of improving lifespan, respiratory, motor, and synaptic function [17], [77]. These studies will need to assess cognitive and social abnormalities to determine whether higher-order processes can be alleviated as well. It is plausable, therefore, that administration of another drug targeted at improving synaptic function in adulthood may be more effective in *Mecp2* mutants that were treated with ALC during early postnatal development.

We show here that ALC attenuates a wider range of early behavioral deficits in *Mecp2* mutants than pharmacological therapies described previously (reviewed in [11], [78]) and with no apparent adverse effects. Most notably, this is the first study to report significant rescue of dendritic morphology in *Mecp2* mutant mice. We hypothesize that ALC's ability to ameliorate a wide range of behavioral and pathological abnormalities is due both to its broad range of action and the early timing of the intervention [32]. We hypothesize that because we intervened during the critical period of synaptic formation and refinement in the cortex, we were able to rescue dendritic abnormalities.

This study provides potential insight into the successes and failures of prior clinical trials administering ALC or L-carnitine to girls with RTT. One of the improvements noted in RTT girls treated with carnitine is an increase in energy levels [28]. Similarly, the only behavioral phenotypes ALC treatment could amelorate past PN 36 in *Mecp2* mutants were the decreases in oxygen consumption and carbon dioxide production. Gas exchange reflects metabolic rate and overall energy expenditure suggesting that ALC-treatment likely enhanced energy production in the null mutants [30], [60], [79]. The increase in energy noted in both RTT girls and our mice, with no concurrent increase in motor activity levels, supports the premise that, in later disease stages, girls with RTT and *Mecp2* mutants respond similarly to ALC.

In clinical trials, treatment with carnitine occurs after symptoms appear and long after critical periods for cortical reorganization are closed. This time may be equivalent to the time in which ALC-treatment has little effect in *Mecp2* mutant mice. This lack of improvement suggests an important mode of action through which carnitine operates, namely on establishing neuronal networks. These studies suggest that there is an early stage in RTT girls, which may be much more ameniable to ALC treatment.

## Conclusions

Our studies using ALC suggest that the most effective therapy for RTT may involve a multi-step pharamacological approach including early treatments to establish functioning neuronal networks, and then later treatments targeted to specific synaptic abnormalities. Administration of ALC in mice in this study began at a developmental period equivalent to the third trimester in humans [80], in other words, before birth and earlier than RTT is currently diagnosed in humans. We speculate that when highly effective treatments become available that can prevent symptom onset in RTT, then genetic screening for MeCP2 mutations and early therapeutic interventions will become available, such is the practice for treating other genetic disorders. Thus, early treatment with ALC appears to be a promising theraputic addition to a future pharmacological approach to treating RTT. This study provides hope that, in conjuction with other drugs, the severe symptoms of RTT can be attenuated.
